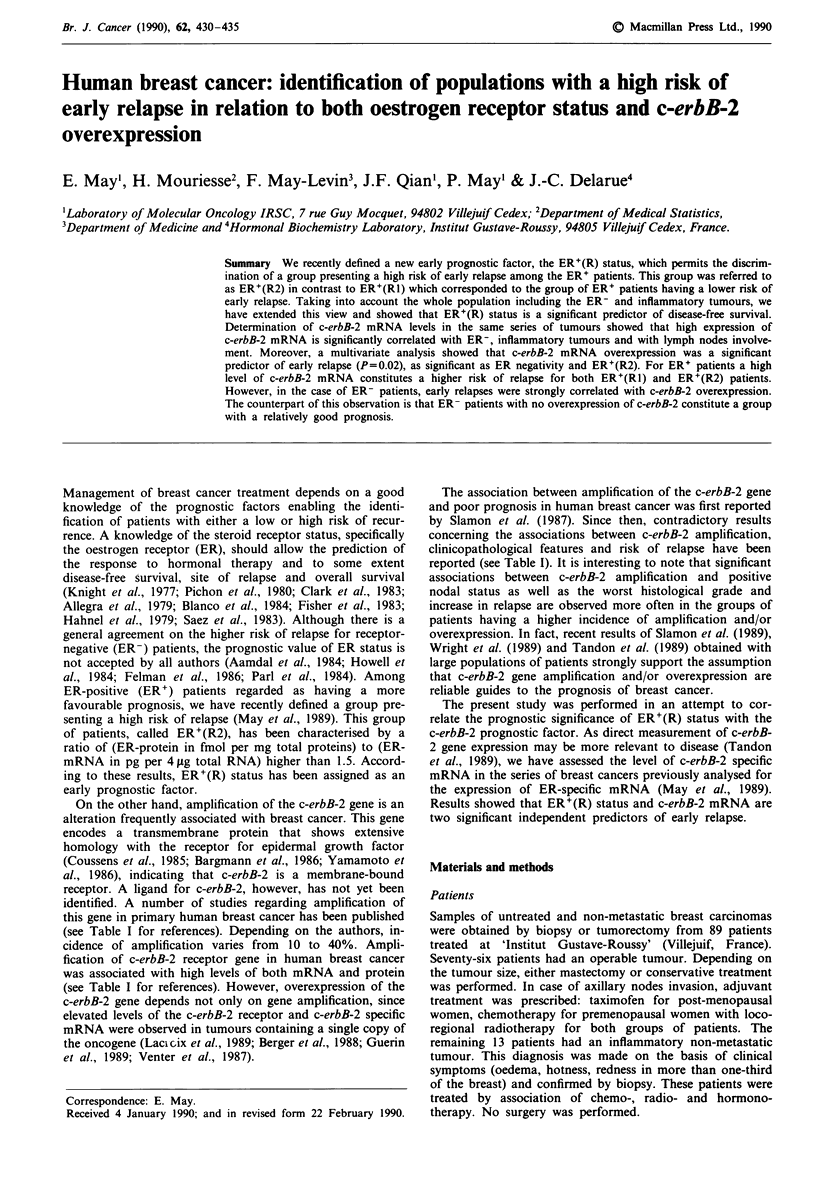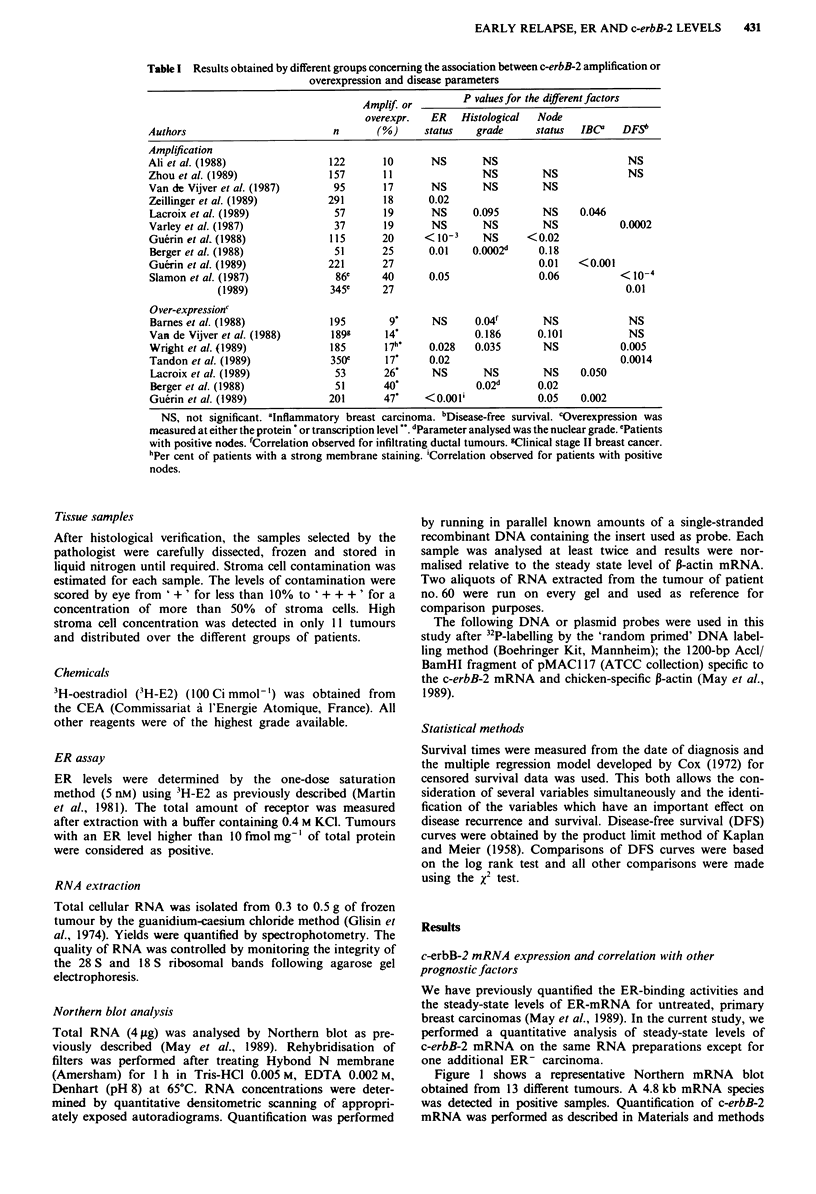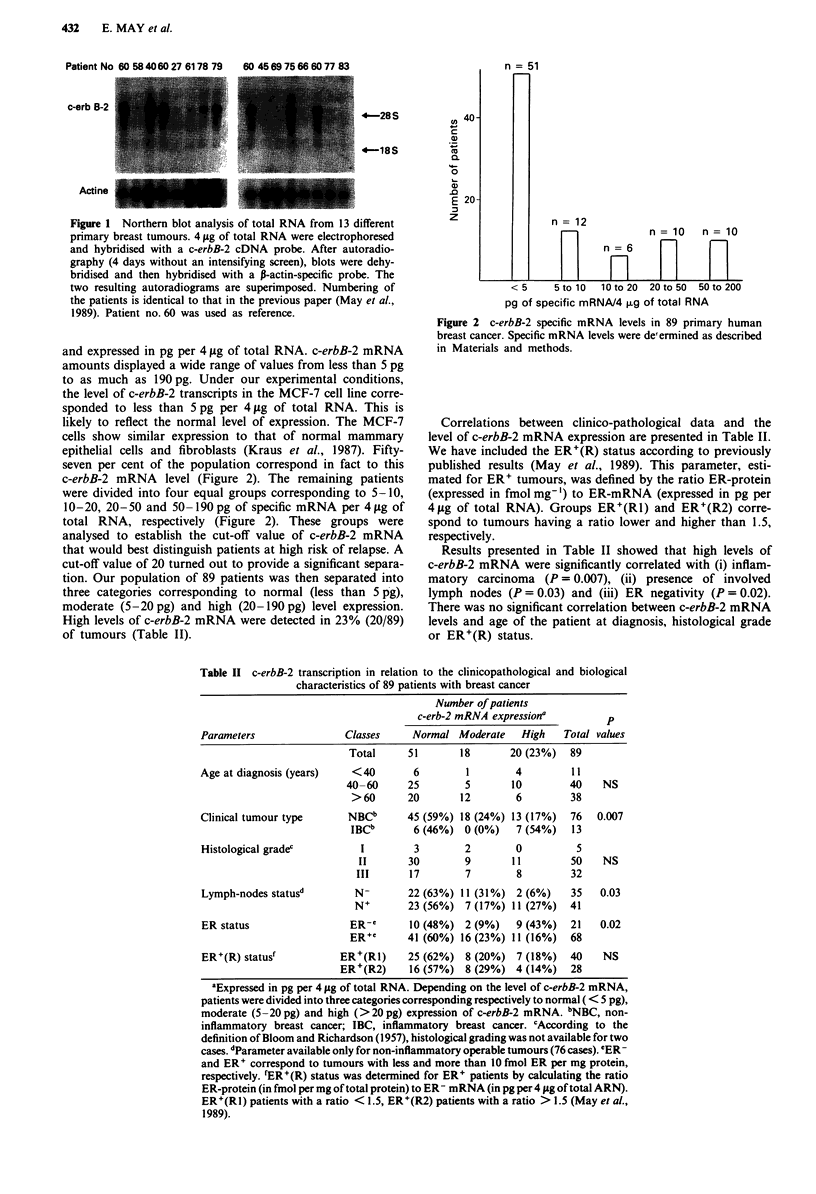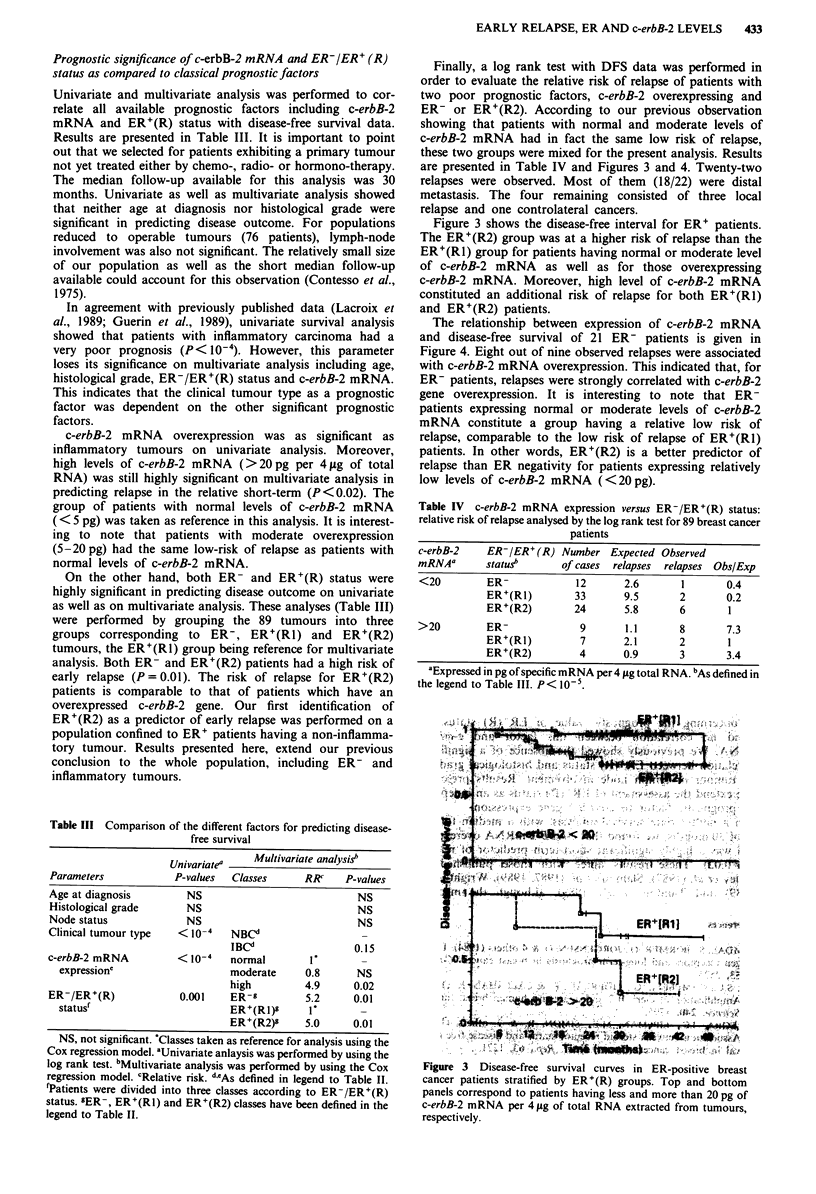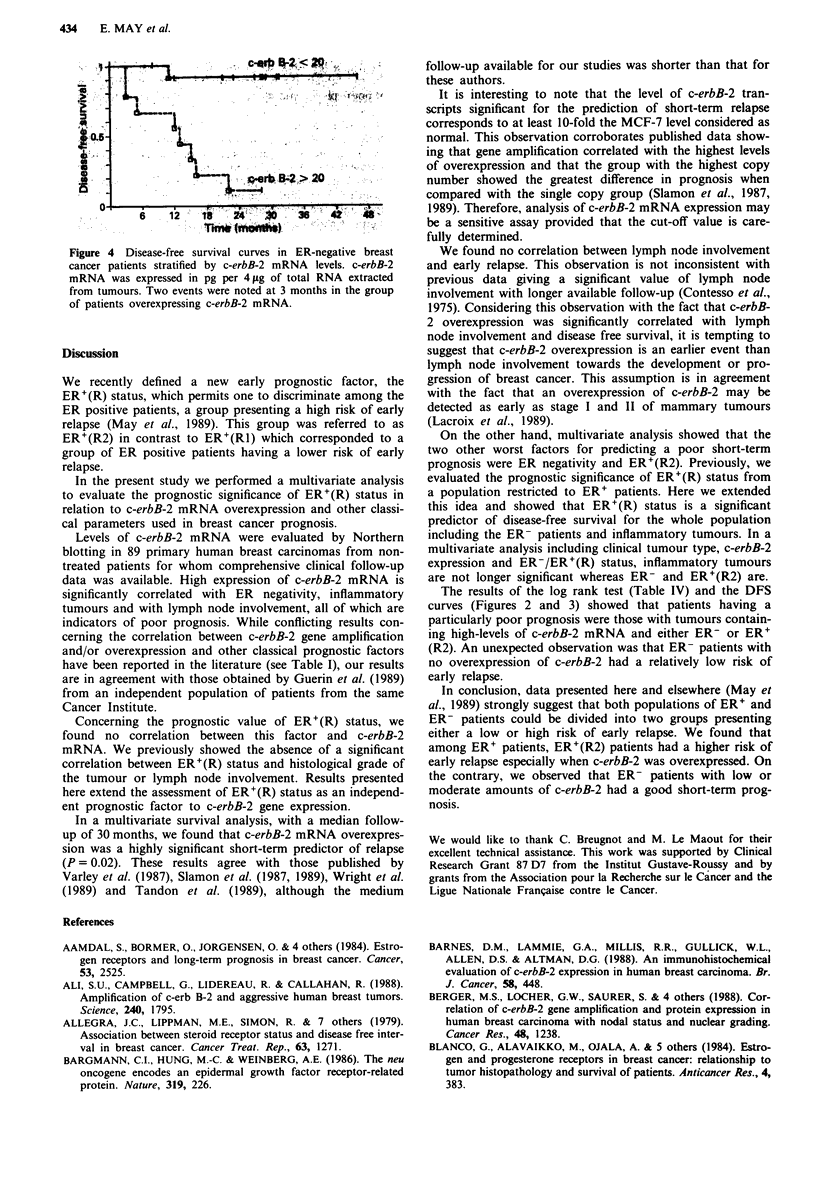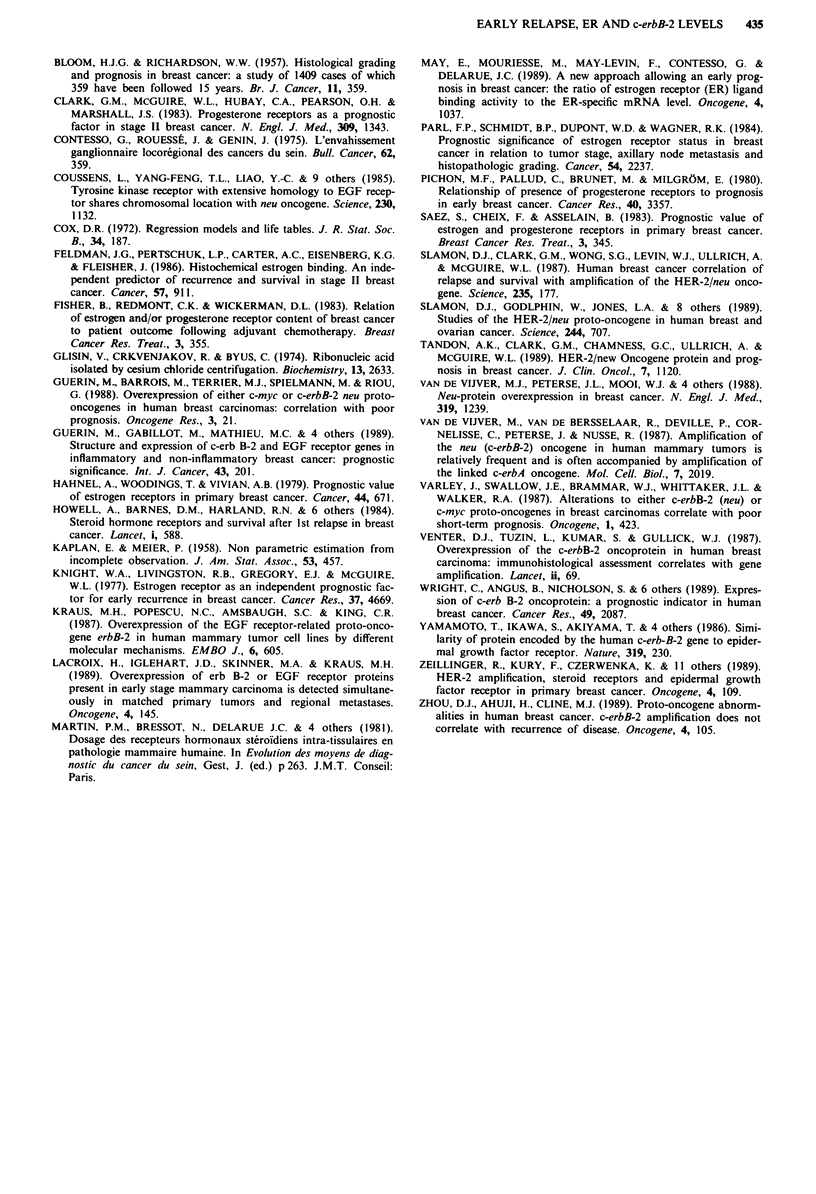# Human breast cancer: identification of populations with a high risk of early relapse in relation to both oestrogen receptor status and c-erbB-2 overexpression.

**DOI:** 10.1038/bjc.1990.312

**Published:** 1990-09

**Authors:** E. May, H. Mouriesse, F. May-Levin, J. F. Qian, P. May, J. C. Delarue

**Affiliations:** Laboratory of Molecular Oncology IRSC, Villejuif, France.

## Abstract

**Images:**


Br. J. Cancer (1990), 62, 430-435 © Macmillan Press Ltd., 1990

### Human breast cancer: identification of populations with a high risk of early relapse in relation to both oestrogen receptor status and c-*erbB*-2 overexpression

E. May[1], H. Mouriesse[2], F. May-Levin[3], J.F. Qian[1], P. May[1] & J.-C. Delarue[4]

[1]*Laboratory of Molecular Oncology IRSC, 7 rue Guy Mocquet, 94802 Villejuif Cedex;* [2]*Department of Medical Statistics,* [3]*Department of Medicine and* [4]*Hormonal Biochemistry Laboratory, Institut Gustave-Roussy, 94805 Villejuif Cedex, France.*

**Summary** We recently defined a new early prognostic factor, the $ER^+$(R) status, which permits the discrimination of a group presenting a high risk of early relapse among the $ER^+$ patients. This group was referred to as $ER^+$(R2) in contrast to $ER^+$(R1) which corresponded to the group of $ER^+$ patients having a lower risk of early relapse. Taking into account the whole population including the $ER^-$ and inflammatory tumours, we have extended this view and showed that $ER^+$(R) status is a significant predictor of disease-free survival. Determination of c-*erbB*-2 mRNA levels in the same series of tumours showed that high expression of c-*erbB*-2 mRNA is significantly correlated with $ER^-$, inflammatory tumours and with lymph nodes involvement. Moreover, a multivariate analysis showed that c-*erbB*-2 mRNA overexpression was a significant predictor of early relapse ($P=0.02$), as significant as ER negativity and $ER^+$(R2). For $ER^+$ patients a high level of c-*erbB*-2 mRNA constitutes a higher risk of relapse for both $ER^+$(R1) and $ER^+$(R2) patients. However, in the case of $ER^-$ patients, early relapses were strongly correlated with c-*erbB*-2 overexpression. The counterpart of this observation is that $ER^-$ patients with no overexpression of c-*erbB*-2 constitute a group with a relatively good prognosis.

Management of breast cancer treatment depends on a good knowledge of the prognostic factors enabling the identification of patients with either a low or high risk of recurrence. A knowledge of the steroid receptor status, specifically the oestrogen receptor (ER), should allow the prediction of the response to hormonal therapy and to some extent disease-free survival, site of relapse and overall survival (Knight *et al.*, 1977; Pichon *et al.*, 1980; Clark *et al.*, 1983; Allegra *et al.*, 1979; Blanco *et al.*, 1984; Fisher *et al.*, 1983; Hahnel *et al.*, 1979; Saez *et al.*, 1983). Although there is a general agreement on the higher risk of relapse for receptor-negative ($ER^-$) patients, the prognostic value of ER status is not accepted by all authors (Aamdal *et al.*, 1984; Howell *et al.*, 1984; Felman *et al.*, 1986; Parl *et al.*, 1984). Among ER-positive ($ER^+$) patients regarded as having a more favourable prognosis, we have recently defined a group presenting a high risk of relapse (May *et al.*, 1989). This group of patients, called $ER^+$(R2), has been characterised by a ratio of (ER-protein in fmol per mg total proteins) to (ER-mRNA in pg per 4 μg total RNA) higher than 1.5. According to these results, $ER^+$(R) status has been assigned as an early prognostic factor.

On the other hand, amplification of the c-*erbB*-2 gene is an alteration frequently associated with breast cancer. This gene encodes a transmembrane protein that shows extensive homology with the receptor for epidermal growth factor (Coussens *et al.*, 1985; Bargmann *et al.*, 1986; Yamamoto *et al.*, 1986), indicating that c-*erbB*-2 is a membrane-bound receptor. A ligand for c-*erbB*-2, however, has not yet been identified. A number of studies regarding amplification of this gene in primary human breast cancer has been published (see Table I for references). Depending on the authors, incidence of amplification varies from 10 to 40%. Amplification of c-*erbB*-2 receptor gene in human breast cancer was associated with high levels of both mRNA and protein (see Table I for references). However, overexpression of the c-*erbB*-2 gene depends not only on gene amplification, since elevated levels of the c-*erbB*-2 receptor and c-*erbB*-2 specific mRNA were observed in tumours containing a single copy of the oncogene (Lacroix *et al.*, 1989; Berger *et al.*, 1988; Guerin *et al.*, 1989; Venter *et al.*, 1987).

Correspondence: E. May.
Received 4 January 1990; and in revised form 22 February 1990.

The association between amplification of the c-*erbB*-2 gene and poor prognosis in human breast cancer was first reported by Slamon *et al.* (1987). Since then, contradictory results concerning the associations between c-*erbB*-2 amplification, clinicopathological features and risk of relapse have been reported (see Table I). It is interesting to note that significant associations between c-*erbB*-2 amplification and positive nodal status as well as the worst histological grade and increase in relapse are observed more often in the groups of patients having a higher incidence of amplification and/or overexpression. In fact, recent results of Slamon *et al.* (1989), Wright *et al.* (1989) and Tandon *et al.* (1989) obtained with large populations of patients strongly support the assumption that c-*erbB*-2 gene amplification and/or overexpression are reliable guides to the prognosis of breast cancer.

The present study was performed in an attempt to correlate the prognostic significance of $ER^+$(R) status with the c-*erbB*-2 prognostic factor. As direct measurement of c-*erbB*-2 gene expression may be more relevant to disease (Tandon *et al.*, 1989), we have assessed the level of c-*erbB*-2 specific mRNA in the series of breast cancers previously analysed for the expression of ER-specific mRNA (May *et al.*, 1989). Results showed that $ER^+$(R) status and c-*erbB*-2 mRNA are two significant independent predictors of early relapse.

### Materials and methods

### *Patients*

Samples of untreated and non-metastatic breast carcinomas were obtained by biopsy or tumorectomy from 89 patients treated at 'Institut Gustave-Roussy' (Villejuif, France). Seventy-six patients had an operable tumour. Depending on the tumour size, either mastectomy or conservative treatment was performed. In case of axillary nodes invasion, adjuvant treatment was prescribed: taximofen for post-menopausal women, chemotherapy for premenopausal women with loco-regional radiotherapy for both groups of patients. The remaining 13 patients had an inflammatory non-metastatic tumour. This diagnosis was made on the basis of clinical symptoms (oedema, hotness, redness in more than one-third of the breast) and confirmed by biopsy. These patients were treated by association of chemo-, radio- and hormonotherapy. No surgery was performed.

**Table I** Results obtained by different groups concerning the association between c-*erbB*-2 amplification or overexpression and disease parameters

| *Authors* | *n* | *Amplif. or overexpr. (%)* | *P values for the different factors* | | | | |
|---|---|---|---|---|---|---|---|
| | | | *ER status* | *Histological grade* | *Node status* | *IBC*[a] | *DFS*[b] |
| *Amplification* | | | | | | | |
| Ali *et al.* (1988) | 122 | 10 | NS | NS | | | NS |
| Zhou *et al.* (1989) | 157 | 11 | | NS | NS | | NS |
| Van de Vijver *et al.* (1987) | 95 | 17 | NS | NS | NS | | |
| Zeillinger *et al.* (1989) | 291 | 18 | 0.02 | | | | |
| Lacroix *et al.* (1989) | 57 | 19 | NS | 0.095 | NS | 0.046 | |
| Varley *et al.* (1987) | 37 | 19 | NS | NS | NS | | 0.0002 |
| Guérin *et al.* (1988) | 115 | 20 | $<10^{-3}$ | NS | <0.02 | | |
| Berger *et al.* (1988) | 51 | 25 | 0.01 | 0.0002[d] | 0.18 | | |
| Guérin *et al.* (1989) | 221 | 27 | | | 0.01 | <0.001 | |
| Slamon *et al.* (1987) | 86[e] | 40 | 0.05 | | 0.06 | | $<10^{-4}$ |
| (1989) | 345[e] | 27 | | | | | 0.01 |
| *Over-expression*[c] | | | | | | | |
| Barnes *et al.* (1988) | 195 | 9* | NS | 0.04[f] | NS | | NS |
| Van de Vijver *et al.* (1988) | 189[g] | 14* | | 0.186 | 0.101 | | NS |
| Wright *et al.* (1989) | 185 | 17[h]* | 0.028 | 0.035 | NS | | 0.005 |
| Tandon *et al.* (1989) | 350[e] | 17* | 0.02 | | | | 0.0014 |
| Lacroix *et al.* (1989) | 53 | 26* | NS | NS | NS | 0.050 | |
| Berger *et al.* (1988) | 51 | 40* | | 0.02[d] | 0.02 | | |
| Guérin *et al.* (1989) | 201 | 47* | <0.001[i] | | 0.05 | 0.002 | |

NS, not significant. [a]Inflammatory breast carcinoma. [b]Disease-free survival. [c]Overexpression was measured at either the protein * or transcription level **. [d]Parameter analysed was the nuclear grade. [e]Patients with positive nodes. [f]Correlation observed for infiltrating ductal tumours. [g]Clinical stage II breast cancer. [h]Per cent of patients with a strong membrane staining. [i]Correlation observed for patients with positive nodes.

### *Tissue samples*

After histological verification, the samples selected by the pathologist were carefully dissected, frozen and stored in liquid nitrogen until required. Stroma cell contamination was estimated for each sample. The levels of contamination were scored by eye from ' + ' for less than 10% to ' + + + ' for a concentration of more than 50% of stroma cells. High stroma cell concentration was detected in only 11 tumours and distributed over the different groups of patients.

### *Chemicals*

$^3$H-oestradiol ($^3$H-E2) (100 Ci mmol$^{-1}$) was obtained from the CEA (Commissariat à l'Energie Atomique, France). All other reagents were of the highest grade available.

### *ER assay*

ER levels were determined by the one-dose saturation method (5 nM) using $^3$H-E2 as previously described (Martin *et al.*, 1981). The total amount of receptor was measured after extraction with a buffer containing 0.4 M KCl. Tumours with an ER level higher than 10 fmol mg$^{-1}$ of total protein were considered as positive.

### *RNA extraction*

Total cellular RNA was isolated from 0.3 to 0.5 g of frozen tumour by the guanidium-caesium chloride method (Glisin *et al.*, 1974). Yields were quantified by spectrophotometry. The quality of RNA was controlled by monitoring the integrity of the 28 S and 18 S ribosomal bands following agarose gel electrophoresis.

### *Northern blot analysis*

Total RNA (4 µg) was analysed by Northern blot as previously described (May *et al.*, 1989). Rehybridisation of filters was performed after treating Hybond N membrane (Amersham) for 1 h in Tris-HCl 0.005 M, EDTA 0.002 M, Denhart (pH 8) at 65°C. RNA concentrations were determined by quantitative densitometric scanning of appropriately exposed autoradiograms. Quantification was performed by running in parallel known amounts of a single-stranded recombinant DNA containing the insert used as probe. Each sample was analysed at least twice and results were normalised relative to the steady state level of β-actin mRNA. Two aliquots of RNA extracted from the tumour of patient no. 60 were run on every gel and used as reference for comparison purposes.

The following DNA or plasmid probes were used in this study after $^{32}$P-labelling by the 'random primed' DNA labelling method (Boehringer Kit, Mannheim); the 1200-bp AccI/BamHI fragment of pMAC117 (ATCC collection) specific to the c-*erbB*-2 mRNA and chicken-specific β-actin (May *et al.*, 1989).

### *Statistical methods*

Survival times were measured from the date of diagnosis and the multiple regression model developed by Cox (1972) for censored survival data was used. This both allows the consideration of several variables simultaneously and the identification of the variables which have an important effect on disease recurrence and survival. Disease-free survival (DFS) curves were obtained by the product limit method of Kaplan and Meier (1958). Comparisons of DFS curves were based on the log rank test and all other comparisons were made using the $\chi^2$ test.

### Results

### c-*erbB*-2 *mRNA expression and correlation with other prognostic factors*

We have previously quantified the ER-binding activities and the steady-state levels of ER-mRNA for untreated, primary breast carcinomas (May *et al.*, 1989). In the current study, we performed a quantitative analysis of steady-state levels of c-*erbB*-2 mRNA on the same RNA preparations except for one additional ER$^-$ carcinoma.

Figure 1 shows a representative Northern mRNA blot obtained from 13 different tumours. A 4.8 kb mRNA species was detected in positive samples. Quantification of c-*erbB*-2 mRNA was performed as described in Materials and methods

**Figure 1** Northern blot analysis of total RNA from 13 different primary breast tumours. 4 μg of total RNA were electrophoresed and hybridised with a c-*erbB*-2 cDNA probe. After autoradiography (4 days without an intensifying screen), blots were dehybridised and then hybridised with a β-actin-specific probe. The two resulting autoradiograms are superimposed. Numbering of the patients is identical to that in the previous paper (May *et al.*, 1989). Patient no. 60 was used as reference.

and expressed in pg per 4 μg of total RNA. c-*erbB*-2 mRNA amounts displayed a wide range of values from less than 5 pg to as much as 190 pg. Under our experimental conditions, the level of c-*erbB*-2 transcripts in the MCF-7 cell line corresponded to less than 5 pg per 4 μg of total RNA. This is likely to reflect the normal level of expression. The MCF-7 cells show similar expression to that of normal mammary epithelial cells and fibroblasts (Kraus *et al.*, 1987). Fifty-seven per cent of the population correspond in fact to this c-*erbB*-2 mRNA level (Figure 2). The remaining patients were divided into four equal groups corresponding to 5–10, 10–20, 20–50 and 50–190 pg of specific mRNA per 4 μg of total RNA, respectively (Figure 2). These groups were analysed to establish the cut-off value of c-*erbB*-2 mRNA that would best distinguish patients at high risk of relapse. A cut-off value of 20 turned out to provide a significant separation. Our population of 89 patients was then separated into three categories corresponding to normal (less than 5 pg), moderate (5–20 pg) and high (20–190 pg) level expression. High levels of c-*erbB*-2 mRNA were detected in 23% (20/89) of tumours (Table II).

**Figure 2** c-*erbB*-2 specific mRNA levels in 89 primary human breast cancer. Specific mRNA levels were de'ermined as described in Materials and methods.

Correlations between clinico-pathological data and the level of c-*erbB*-2 mRNA expression are presented in Table II. We have included the $ER^+$(R) status according to previously published results (May *et al.*, 1989). This parameter, estimated for $ER^+$ tumours, was defined by the ratio ER-protein (expressed in fmol $mg^{-1}$) to ER-mRNA (expressed in pg per 4 μg of total RNA). Groups $ER^+$(R1) and $ER^+$(R2) correspond to tumours having a ratio lower and higher than 1.5, respectively.

Results presented in Table II showed that high levels of c-*erbB*-2 mRNA were significantly correlated with (i) inflammatory carcinoma ($P = 0.007$), (ii) presence of involved lymph nodes ($P = 0.03$) and (iii) ER negativity ($P = 0.02$). There was no significant correlation between c-*erbB*-2 mRNA levels and age of the patient at diagnosis, histological grade or $ER^+$(R) status.

**Table II** c-*erbB*-2 transcription in relation to the clinicopathological and biological characteristics of 89 patients with breast cancer

| *Parameters* | *Classes* | *Number of patients c-erb-2 mRNA expression*[a] *Normal* | *Moderate* | *High* | *Total* | *P values* |
|---|---|---|---|---|---|---|
| | Total | 51 | 18 | 20 (23%) | 89 | |
| Age at diagnosis (years) | <40 | 6 | 1 | 4 | 11 | |
| | 40–60 | 25 | 5 | 10 | 40 | NS |
| | >60 | 20 | 12 | 6 | 38 | |
| Clinical tumour type | NBC[b] | 45 (59%) | 18 (24%) | 13 (17%) | 76 | 0.007 |
| | IBC[b] | 6 (46%) | 0 (0%) | 7 (54%) | 13 | |
| Histological grade[c] | I | 3 | 2 | 0 | 5 | |
| | II | 30 | 9 | 11 | 50 | NS |
| | III | 17 | 7 | 8 | 32 | |
| Lymph-nodes status[d] | $N^-$ | 22 (63%) | 11 (31%) | 2 (6%) | 35 | 0.03 |
| | $N^+$ | 23 (56%) | 7 (17%) | 11 (27%) | 41 | |
| ER status | $ER^-$[e] | 10 (48%) | 2 (9%) | 9 (43%) | 21 | 0.02 |
| | $ER^+$[e] | 41 (60%) | 16 (23%) | 11 (16%) | 68 | |
| $ER^+$(R) status[f] | $ER^+$(R1) | 25 (62%) | 8 (20%) | 7 (18%) | 40 | NS |
| | $ER^+$(R2) | 16 (57%) | 8 (29%) | 4 (14%) | 28 | |

[a]Expressed in pg per 4 μg of total RNA. Depending on the level of c-*erbB*-2 mRNA, patients were divided into three categories corresponding respectively to normal (< 5 pg), moderate (5–20 pg) and high (> 20 pg) expression of c-*erbB*-2 mRNA. [b]NBC, non-inflammatory breast cancer; IBC, inflammatory breast cancer. [c]According to the definition of Bloom and Richardson (1957), histological grading was not available for two cases. [d]Parameter available only for non-inflammatory operable tumours (76 cases). [e]$ER^-$ and $ER^+$ correspond to tumours with less and more than 10 fmol ER per mg protein, respectively. [f]$ER^+$(R) status was determined for $ER^+$ patients by calculating the ratio ER-protein (in fmol per mg of total protein) to $ER^-$ mRNA (in pg per 4 μg of total ARN). $ER^+$(R1) patients with a ratio < 1.5, $ER^+$(R2) patients with a ratio > 1.5 (May *et al.*, 1989).

*Prognostic significance of c-*erbB*-2 mRNA and $ER^-/ER^+$ (R) status as compared to classical prognostic factors*

Univariate and multivariate analysis was performed to correlate all available prognostic factors including c-*erbB*-2 mRNA and $ER^+$(R) status with disease-free survival data. Results are presented in Table III. It is important to point out that we selected for patients exhibiting a primary tumour not yet treated either by chemo-, radio- or hormono-therapy. The median follow-up available for this analysis was 30 months. Univariate as well as multivariate analysis showed that neither age at diagnosis nor histological grade were significant in predicting disease outcome. For populations reduced to operable tumours (76 patients), lymph-node involvement was also not significant. The relatively small size of our population as well as the short median follow-up available could account for this observation (Contesso *et al.*, 1975).

In agreement with previously published data (Lacroix *et al.*, 1989; Guerin *et al.*, 1989), univariate survival analysis showed that patients with inflammatory carcinoma had a very poor prognosis ($P < 10^{-4}$). However, this parameter loses its significance on multivariate analysis including age, histological grade, $ER^-/ER^+$(R) status and c-*erbB*-2 mRNA. This indicates that the clinical tumour type as a prognostic factor was dependent on the other significant prognostic factors.

c-*erbB*-2 mRNA overexpression was as significant as inflammatory tumours on univariate analysis. Moreover, high levels of c-*erbB*-2 mRNA (>20 pg per 4 µg of total RNA) was still highly significant on multivariate analysis in predicting relapse in the relative short-term ($P < 0.02$). The group of patients with normal levels of c-*erbB*-2 mRNA (<5 pg) was taken as reference in this analysis. It is interesting to note that patients with moderate overexpression (5–20 pg) had the same low-risk of relapse as patients with normal levels of c-*erbB*-2 mRNA.

On the other hand, both $ER^-$ and $ER^+$(R) status were highly significant in predicting disease outcome on univariate as well as on multivariate analysis. These analyses (Table III) were performed by grouping the 89 tumours into three groups corresponding to $ER^-$, $ER^+$(R1) and $ER^+$(R2) tumours, the $ER^+$(R1) group being reference for multivariate analysis. Both $ER^-$ and $ER^+$(R2) patients had a high risk of early relapse ($P = 0.01$). The risk of relapse for $ER^+$(R2) patients is comparable to that of patients which have an overexpressed c-*erbB*-2 gene. Our first identification of $ER^+$(R2) as a predictor of early relapse was performed on a population confined to $ER^+$ patients having a non-inflammatory tumour. Results presented here, extend our previous conclusion to the whole population, including $ER^-$ and inflammatory tumours.

**Table III** Comparison of the different factors for predicting disease-free survival

| Parameters | Univariate[a] P-values | Multivariate analysis[b] Classes | RR[c] | P-values |
|---|---|---|---|---|
| Age at diagnosis | NS | | | NS |
| Histological grade | NS | | | NS |
| Node status | NS | | | NS |
| Clinical tumour type | $<10^{-4}$ | NBC[d] | | – |
| | | IBC[d] | | 0.15 |
| c-*erbB*-2 mRNA expression[e] | $<10^{-4}$ | normal | 1* | – |
| | | moderate | 0.8 | NS |
| | | high | 4.9 | 0.02 |
| $ER^-/ER^+$(R) status[f] | 0.001 | $ER^-$[g] | 5.2 | 0.01 |
| | | $ER^+$(R1)[g] | 1* | – |
| | | $ER^+$(R2)[g] | 5.0 | 0.01 |

NS, not significant. *Classes taken as reference for analysis using the Cox regression model. [a]Univariate anlaysis was performed by using the log rank test. [b]Multivariate analysis was performed by using the Cox regression model. [c]Relative risk. [d,e]As defined in legend to Table II. [f]Patients were divided into three classes according to $ER^-/ER^+$(R) status. [g]$ER^-$, $ER^+$(R1) and $ER^+$(R2) classes have been defined in the legend to Table II.

Finally, a log rank test with DFS data was performed in order to evaluate the relative risk of relapse of patients with two poor prognostic factors, c-*erbB*-2 overexpressing and $ER^-$ or $ER^+$(R2). According to our previous observation showing that patients with normal and moderate levels of c-*erbB*-2 mRNA had in fact the same low risk of relapse, these two groups were mixed for the present analysis. Results are presented in Table IV and Figures 3 and 4. Twenty-two relapses were observed. Most of them (18/22) were distal metastasis. The four remaining consisted of three local relapse and one controlateral cancers.

Figure 3 shows the disease-free interval for $ER^+$ patients. The $ER^+$(R2) group was at a higher risk of relapse than the $ER^+$(R1) group for patients having normal or moderate level of c-*erbB*-2 mRNA as well as for those overexpressing c-*erbB*-2 mRNA. Moreover, high level of c-*erbB*-2 mRNA constituted an additional risk of relapse for both $ER^+$(R1) and $ER^+$(R2) patients.

The relationship between expression of c-*erbB*-2 mRNA and disease-free survival of 21 $ER^-$ patients is given in Figure 4. Eight out of nine observed relapses were associated with c-*erbB*-2 mRNA overexpression. This indicated that, for $ER^-$ patients, relapses were strongly correlated with c-*erbB*-2 gene overexpression. It is interesting to note that $ER^-$ patients expressing normal or moderate levels of c-*erbB*-2 mRNA constitute a group having a relative low risk of relapse, comparable to the low risk of relapse of $ER^+$(R1) patients. In other words, $ER^+$(R2) is a better predictor of relapse than ER negativity for patients expressing relatively low levels of c-*erbB*-2 mRNA (<20 pg).

**Table IV** c-*erbB*-2 mRNA expression *versus* $ER^-/ER^+$(R) status: relative risk of relapse analysed by the log rank test for 89 breast cancer patients

| c-*erbB*-2 mRNA[a] | $ER^-/ER^+$(R) status[b] | Number of cases | Expected relapses | Observed relapses | Obs/Exp |
|---|---|---|---|---|---|
| <20 | $ER^-$ | 12 | 2.6 | 1 | 0.4 |
| | $ER^+$(R1) | 33 | 9.5 | 2 | 0.2 |
| | $ER^+$(R2) | 24 | 5.8 | 6 | 1 |
| >20 | $ER^-$ | 9 | 1.1 | 8 | 7.3 |
| | $ER^+$(R1) | 7 | 2.1 | 2 | 1 |
| | $ER^+$(R2) | 4 | 0.9 | 3 | 3.4 |

[a]Expressed in pg of specific mRNA per 4 µg total RNA. [b]As defined in the legend to Table III. $P < 10^{-5}$.

**Figure 3** Disease-free survival curves in ER-positive breast cancer patients stratified by $ER^+$(R) groups. Top and bottom panels correspond to patients having less and more than 20 pg of c-*erbB*-2 mRNA per 4 µg of total RNA extracted from tumours, respectively.

**Figure 4** Disease-free survival curves in ER-negative breast cancer patients stratified by c-*erbB*-2 mRNA levels. c-*erbB*-2 mRNA was expressed in pg per 4 μg of total RNA extracted from tumours. Two events were noted at 3 months in the group of patients overexpressing c-*erbB*-2 mRNA.

### Discussion

We recently defined a new early prognostic factor, the $ER^+$(R) status, which permits one to discriminate among the ER positive patients, a group presenting a high risk of early relapse (May *et al.*, 1989). This group was referred to as $ER^+$(R2) in contrast to $ER^+$(R1) which corresponded to a group of ER positive patients having a lower risk of early relapse.

In the present study we performed a multivariate analysis to evaluate the prognostic significance of $ER^+$(R) status in relation to c-*erbB*-2 mRNA overexpression and other classical parameters used in breast cancer prognosis.

Levels of c-*erbB*-2 mRNA were evaluated by Northern blotting in 89 primary human breast carcinomas from non-treated patients for whom comprehensive clinical follow-up data was available. High expression of c-*erbB*-2 mRNA is significantly correlated with ER negativity, inflammatory tumours and with lymph node involvement, all of which are indicators of poor prognosis. While conflicting results concerning the correlation between c-*erbB*-2 gene amplification and/or overexpression and other classical prognostic factors have been reported in the literature (see Table I), our results are in agreement with those obtained by Guerin *et al.* (1989) from an independent population of patients from the same Cancer Institute.

Concerning the prognostic value of $ER^+$(R) status, we found no correlation between this factor and c-*erbB*-2 mRNA. We previously showed the absence of a significant correlation between $ER^+$(R) status and histological grade of the tumour or lymph node involvement. Results presented here extend the assessment of $ER^+$(R) status as an independent prognostic factor to c-*erbB*-2 gene expression.

In a multivariate survival analysis, with a median follow-up of 30 months, we found that c-*erbB*-2 mRNA overexpression was a highly significant short-term predictor of relapse ($P = 0.02$). These results agree with those published by Varley *et al.* (1987), Slamon *et al.* (1987, 1989), Wright *et al.* (1989) and Tandon *et al.* (1989), although the medium follow-up available for our studies was shorter than that for these authors.

It is interesting to note that the level of c-*erbB*-2 transcripts significant for the prediction of short-term relapse corresponds to at least 10-fold the MCF-7 level considered as normal. This observation corroborates published data showing that gene amplification correlated with the highest levels of overexpression and that the group with the highest copy number showed the greatest difference in prognosis when compared with the single copy group (Slamon *et al.*, 1987, 1989). Therefore, analysis of c-*erbB*-2 mRNA expression may be a sensitive assay provided that the cut-off value is carefully determined.

We found no correlation between lymph node involvement and early relapse. This observation is not inconsistent with previous data giving a significant value of lymph node involvement with longer available follow-up (Contesso *et al.*, 1975). Considering this observation with the fact that c-*erbB*-2 overexpression was significantly correlated with lymph node involvement and disease free survival, it is tempting to suggest that c-*erbB*-2 overexpression is an earlier event than lymph node involvement towards the development or progression of breast cancer. This assumption is in agreement with the fact that an overexpression of c-*erbB*-2 may be detected as early as stage I and II of mammary tumours (Lacroix *et al.*, 1989).

On the other hand, multivariate analysis showed that the two other worst factors for predicting a poor short-term prognosis were ER negativity and $ER^+$(R2). Previously, we evaluated the prognostic significance of $ER^+$(R) status from a population restricted to $ER^+$ patients. Here we extended this idea and showed that $ER^+$(R) status is a significant predictor of disease-free survival for the whole population including the $ER^-$ patients and inflammatory tumours. In a multivariate analysis including clinical tumour type, c-*erbB*-2 expression and $ER^-/ER^+$(R) status, inflammatory tumours are not longer significant whereas $ER^-$ and $ER^+$(R2) are.

The results of the log rank test (Table IV) and the DFS curves (Figures 2 and 3) showed that patients having a particularly poor prognosis were those with tumours containing high-levels of c-*erbB*-2 mRNA and either $ER^-$ or $ER^+$ (R2). An unexpected observation was that $ER^-$ patients with no overexpression of c-*erbB*-2 had a relatively low risk of early relapse.

In conclusion, data presented here and elsewhere (May *et al.*, 1989) strongly suggest that both populations of $ER^+$ and $ER^-$ patients could be divided into two groups presenting either a low or high risk of early relapse. We found that among $ER^+$ patients, $ER^+$(R2) patients had a higher risk of early relapse especially when c-*erbB*-2 was overexpressed. On the contrary, we observed that $ER^-$ patients with low or moderate amounts of c-*erbB*-2 had a good short-term prognosis.

We would like to thank C. Breugnot and M. Le Maout for their excellent technical assistance. This work was supported by Clinical Research Grant 87 D7 from the Institut Gustave-Roussy and by grants from the Association pour la Recherche sur le Cancer and the Ligue Nationale Française contre le Cancer.

### References

AAMDAL, S., BORMER, O., JORGENSEN, O. & 4 others (1984). Estrogen receptors and long-term prognosis in breast cancer. *Cancer*, **53**, 2525.

ALI, S.U., CAMPBELL, G., LIDEREAU, R. & CALLAHAN, R. (1988). Amplification of c-erb B-2 and aggressive human breast tumors. *Science*, **240**, 1795.

ALLEGRA, J.C., LIPPMAN, M.E., SIMON, R. & 7 others (1979). Association between steroid receptor status and disease free interval in breast cancer. *Cancer Treat. Rep.*, **63**, 1271.

BARGMANN, C.I., HUNG, M.-C. & WEINBERG, A.E. (1986). The *neu* oncogene encodes an epidermal growth factor receptor-related protein. *Nature*, **319**, 226.

BARNES, D.M., LAMMIE, G.A., MILLIS, R.R., GULLICK, W.L., ALLEN, D.S. & ALTMAN, D.G. (1988). An immunohistochemical evaluation of c-*erbB*-2 expression in human breast carcinoma. *Br. J. Cancer*, **58**, 448.

BERGER, M.S., LOCHER, G.W., SAURER, S. & 4 others (1988). Correlation of c-*erbB*-2 gene amplification and protein expression in human breast carcinoma with nodal status and nuclear grading. *Cancer Res.*, **48**, 1238.

BLANCO, G., ALAVAIKKO, M., OJALA, A. & 5 others (1984). Estrogen and progesterone receptors in breast cancer: relationship to tumor histopathology and survival of patients. *Anticancer Res.*, **4**, 383.

BLOOM, H.J.G. & RICHARDSON, W.W. (1957). Histological grading and prognosis in breast cancer: a study of 1409 cases of which 359 have been followed 15 years. *Br. J. Cancer*, **11**, 359.

CLARK, G.M., MCGUIRE, W.L., HUBAY, C.A., PEARSON, O.H. & MARSHALL, J.S. (1983). Progesterone receptors as a prognostic factor in stage II breast cancer. *N. Engl. J. Med.*, **309**, 1343.

CONTESSO, G., ROUESSÉ, J. & GENIN, J. (1975). L'envahissement ganglionnaire locorégional des cancers du sein. *Bull. Cancer*, **62**, 359.

COUSSENS, L., YANG-FENG, T.L., LIAO, Y.-C. & 9 others (1985). Tyrosine kinase receptor with extensive homology to EGF receptor shares chromosomal location with *neu* oncogene. *Science*, **230**, 1132.

COX, D.R. (1972). Regression models and life tables. *J. R. Stat. Soc. B.*, **34**, 187.

FELDMAN, J.G., PERTSCHUK, L.P., CARTER, A.C., EISENBERG, K.G. & FLEISHER, J. (1986). Histochemical estrogen binding. An independent predictor of recurrence and survival in stage II breast cancer. *Cancer*, **57**, 911.

FISHER, B., REDMONT, C.K. & WICKERMAN, D.L. (1983). Relation of estrogen and/or progesterone receptor content of breast cancer to patient outcome following adjuvant chemotherapy. *Breast Cancer Res. Treat.*, **3**, 355.

GLISIN, V., CRKVENJAKOV, R. & BYUS, C. (1974). Ribonucleic acid isolated by cesium chloride centrifugation. *Biochemistry*, **13**, 2633.

GUERIN, M., BARROIS, M., TERRIER, M.J., SPIELMANN, M. & RIOU, G. (1988). Overexpression of either c-*myc* or c-*erbB*-2 *neu* proto-oncogenes in human breast carcinomas: correlation with poor prognosis. *Oncogene Res.*, **3**, 21.

GUERIN, M., GABILLOT, M., MATHIEU, M.C. & 4 others (1989). Structure and expression of c-erb B-2 and EGF receptor genes in inflammatory and non-inflammatory breast cancer: prognostic significance. *Int. J. Cancer*, **43**, 201.

HAHNEL, A., WOODINGS, T. & VIVIAN, A.B. (1979). Prognostic value of estrogen receptors in primary breast cancer. *Cancer*, **44**, 671.

HOWELL, A., BARNES, D.M., HARLAND, R.N. & 6 others (1984). Steroid hormone receptors and survival after 1st relapse in breast cancer. *Lancet*, **i**, 588.

KAPLAN, E. & MEIER, P. (1958). Non parametric estimation from incomplete observation. *J. Am. Stat. Assoc.*, **53**, 457.

KNIGHT, W.A., LIVINGSTON, R.B., GREGORY, E.J. & MCGUIRE, W.L. (1977). Estrogen receptor as an independent prognostic factor for early recurrence in breast cancer. *Cancer Res.*, **37**, 4669.

KRAUS, M.H., POPESCU, N.C., AMSBAUGH, S.C. & KING, C.R. (1987). Overexpression of the EGF receptor-related proto-oncogene *erbB*-2 in human mammary tumor cell lines by different molecular mechanisms. *EMBO J.*, **6**, 605.

LACROIX, H., IGLEHART, J.D., SKINNER, M.A. & KRAUS, M.H. (1989). Overexpression of erb B-2 or EGF receptor proteins present in early stage mammary carcinoma is detected simultaneously in matched primary tumors and regional metastases. *Oncogene*, **4**, 145.

MARTIN, P.M., BRESSOT, N., DELARUE J.C. & 4 others (1981). Dosage des recepteurs hormonaux stéroïdiens intra-tissulaires en pathologie mammaire humaine. In *Evolution des moyens de diagnostic du cancer du sein*, Gest, J. (ed.) p 263. J.M.T. Conseil: Paris.

MAY, E., MOURIESSE, M., MAY-LEVIN, F., CONTESSO, G. & DELARUE, J.C. (1989). A new approach allowing an early prognosis in breast cancer: the ratio of estrogen receptor (ER) ligand binding activity to the ER-specific mRNA level. *Oncogene*, **4**, 1037.

PARL, F.P., SCHMIDT, B.P., DUPONT, W.D. & WAGNER, R.K. (1984). Prognostic significance of estrogen receptor status in breast cancer in relation to tumor stage, axillary node metastasis and histopathologic grading. *Cancer*, **54**, 2237.

PICHON, M.F., PALLUD, C., BRUNET, M. & MILGRÖM, E. (1980). Relationship of presence of progesterone receptors to prognosis in early breast cancer. *Cancer Res.*, **40**, 3357.

SAEZ, S., CHEIX, F. & ASSELAIN, B. (1983). Prognostic value of estrogen and progesterone receptors in primary breast cancer. *Breast Cancer Res. Treat.*, **3**, 345.

SLAMON, D.J., CLARK, G.M., WONG, S.G., LEVIN, W.J., ULLRICH, A. & MCGUIRE, W.L. (1987). Human breast cancer correlation of relapse and survival with amplification of the HER-2/*neu* oncogene. *Science*, **235**, 177.

SLAMON, D.J., GODLPHIN, W., JONES, L.A. & 8 others (1989). Studies of the HER-2/*neu* proto-oncogene in human breast and ovarian cancer. *Science*, **244**, 707.

TANDON, A.K., CLARK, G.M., CHAMNESS, G.C., ULLRICH, A. & MCGUIRE, W.L. (1989). HER-2/new Oncogene protein and prognosis in breast cancer. *J. Clin. Oncol.*, **7**, 1120.

VAN DE VIJVER, M.J., PETERSE, J.L., MOOI, W.J. & 4 others (1988). *Neu*-protein overexpression in breast cancer. *N. Engl. J. Med.*, **319**, 1239.

VAN DE VIJVER, M., VAN DE BERSSELAAR, R., DEVILLE, P., CORNELISSE, C., PETERSE, J. & NUSSE, R. (1987). Amplification of the *neu* (c-*erbB*-2) oncogene in human mammary tumors is relatively frequent and is often accompanied by amplification of the linked c-*erbA* oncogene. *Mol. Cell. Biol.*, **7**, 2019.

VARLEY, J., SWALLOW, J.E., BRAMMAR, W.J., WHITTAKER, J.L. & WALKER, R.A. (1987). Alterations to either c-*erb*B-2 (*neu*) or c-*myc* proto-oncogenes in breast carcinomas correlate with poor short-term prognosis. *Oncogene*, **1**, 423.

VENTER, D.J., TUZIN, L., KUMAR, S. & GULLICK, W.J. (1987). Overexpression of the c-*erb*B-2 oncoprotein in human breast carcinoma: immunohistological assessment correlates with gene amplification. *Lancet*, **ii**, 69.

WRIGHT, C., ANGUS, B., NICHOLSON, S. & 6 others (1989). Expression of c-*erb* B-2 oncoprotein: a prognostic indicator in human breast cancer. *Cancer Res.*, **49**, 2087.

YAMAMOTO, T., IKAWA, S., AKIYAMA, T. & 4 others (1986). Similarity of protein encoded by the human c-*erb*-*B*-2 gene to epidermal growth factor receptor. *Nature*, **319**, 230.

ZEILLINGER, R., KURY, F., CZERWENKA, K. & 11 others (1989). HER-2 amplification, steroid receptors and epidermal growth factor receptor in primary breast cancer. *Oncogene*, **4**, 109.

ZHOU, D.J., AHUJI, H., CLINE, M.J. (1989). Proto-oncogene abnormalities in human breast cancer. c-*erbB*-2 amplification does not correlate with recurrence of disease. *Oncogene*, **4**, 105.